# Isolation and Characterization of Lytic Phages Infecting Clinical *Klebsiella pneumoniae* from Tunisia

**DOI:** 10.3390/antibiotics13121154

**Published:** 2024-12-02

**Authors:** Donia Mourali, Rahma Kazdaghli, Marwa Gara-Ali, Houda Ben-Miled, Lucas Mora-Quilis, Pilar Domingo-Calap, Kamel Ben-Mahrez

**Affiliations:** 1 Biochemistry and Biotechnology Laboratory LR01ES05, Faculty of Sciences of Tunis, University of Tunis El Manar, El Manar II, Tunis 2092, Tunisia; mouralidonia0@gmail.com (D.M.); kazdaghlirahma01@gmail.com (R.K.); garaalimarwa@gmail.com (M.G.-A.); houda_b.miled@hotmail.fr (H.B.-M.); 2 Institute for Integrative Systems Biology, I2SysBio, Universitat de Valencia-CSIC, 46980 Paterna, Spain

**Keywords:** *Klebsiella pneumoniae*, phage isolation, host range, stability, lysis, comparative genomics

## Abstract

**Background**: *Klebsiella pneumoniae* is an opportunistic pathogen that causes a wide range of infections worldwide. The emergence and spread of multidrug-resistant clones requires the implementation of novel therapeutics, and phages are a promising approach. **Results**: In this study, two *Klebsiella* phages, KpTDp1 and KpTDp2, were isolated from wastewater samples in Tunisia. These phages had a narrow host range and specifically targeted the hypervirulent K2 and K28 capsular types of *K. pneumoniae*. Both phages have double-stranded linear DNA genomes of 49,311 and 49,084 bp, respectively. Comparative genomic and phylogenetic analyses placed phage KpTDp2 in the genus *Webervirus*, while phage KpTDp1 showed some homology with members of the genus *Jedunavirus*, although its placement in a new undescribed genus may be reconsidered. The replication efficiency and lytic ability of these phages, combined with their high stability at temperatures up to 70 °C and pH values ranging from 3.5 to 8.2, highlight the potential of these phages as good candidates for the control of hypervirulent multidrug-resistant *K. pneumoniae*. **Methods**: Phage isolation, titration and multiplicity of infection were performed. The stability of KpTDp1 and KpTDp2 was tested at different pH and temperatures. Genomic characterization was done by genome sequencing, annotation and phylogenetic analysis. **Conclusions**: The ability of KpTDp1 and KpTDp2 to lyse one of the most virulent serotypes of *K. pneumoniae*, as well as the stability of their lytic activities to pH and temperature variations, make these phages promising candidates for antibacterial control.

## 1. Introduction

*Klebsiella pneumoniae* is a Gram-negative, non-motile, encapsulated bacillus. The capsule is a crucial virulence factor with high diversity, being described more than a hundred different capsular types [1,2]. It is a ubiquitous bacterium found as a commensal microorganism in the digestive and respiratory tracts of animals, including humans, and is also found in various environmental niches. *K. pneumoniae* is frequently identified as the most common cause of nosocomial pneumonia and is associated with a wide range of other infections, including septicemia, urinary tract infections, soft tissue infections, bacteremia, and meningitis [1,3]. *K. pneumoniae* is a member of the ESKAPE group, which includes *Enterococcus faecium*, *Staphylococcus aureus*, *K. pneumoniae*, *Acinetobacter baumannii*, *Pseudomonas aeruginosa*, and *Enterobacter sp.*, pathogens constituting the main cause of nosocomial infections worldwide and generally exhibiting multidrug resistance (MDR) [4,5].

The extensive use of antibiotics has led to the emergence of MDR organisms, which constitutes a serious threat to human health. *K. pneumoniae* exhibits resistance to multiple classes of antibiotics, including β-lactams, carbapenems, and aminoglycosides [6]. Carbapenem-resistant *K. pneumoniae* (CRKP) are tightly associated with high morbidity and mortality rates [7,8]. The most common resistance mechanisms in *K. pneumoniae* include the production of extended-spectrum β-lactamases (ESBLs)—mostly carbapenemases, through the overexpression of efflux pumps—and modification of outer membrane porins [9]. In addition, the global dissemination of these strains is further exacerbated by the horizontal transfer of resistance genes via mobile genetic elements, such as plasmids, transposons, and integrons [10]. This growing crisis underscores the urgent need for alternative therapeutic approaches.

One of the most promising alternatives to the traditional use of antibiotics is phage therapy. Bacteriophages, or phages, are viruses that specifically infect and lyse bacterial cells. Unlike antibiotics, phages exhibit high specificity, reducing the risk of dysbiosis. This allows for the eradication of pathogenic bacteria while preserving the commensal microbiota. Phages are widely present in nature and exhibit great diversity, making nature an excellent reservoir for the isolation of new phages with therapeutic potential. Phage banks are key tools for the storage and classification of well-characterized phages, ensuring their availability for rapid use. In order to increase the number of hosts to be targeted, phage cocktails are routinely used. This involves the use of two or more phages with different host tropism, enabling tailored combinations of phages for specific treatments [11]. The use of biological replicating entities presents regulatory challenges. However, phage-derived proteins such as depolymerases and endolysins offer an alternative to avoid these issues [12,13]. Therefore, phage therapy represents an optimistic and viable strategy to address the global crisis of antibiotic resistance.

In Tunisia, hospitals are facing challenges from nosocomial infections and the rise of MDR bacteria, particularly the emergence of CRKP [14,15]. However, there has been limited research on the isolation of viruses for phage therapy targeting clinical *K. pneumoniae isolates* [16]. This study describes the isolation and the characterization of two novel phages KpTDp1 and KpTDp2 isolated from wastewater targeting a clinical strain of *K. pneumoniae* (Kp704) at the Biology Department of Ben Arous Regional Hospital, Tunisia.

## 2. Results

### 2.1. Isolation and Purification of Phages KpTDp1 and KpTDp2

The aim of this study was to isolate and characterize lytic phages targeting a clinical strain of *K. pneumoniae* from wastewater samples collected in Tunis (Tunisia). The clinical isolate Kp704, identified as *K. pneumoniae* via PCR amplification of the 16S rRNA gene, was used as the host strain. The exposure of Kp704 to these samples led to the isolation of two phages, named KpTDp1 and KpTDp2.

### 2.2. Plaque Morphology: Similar Morphology, Different Size

**Figure 1:**
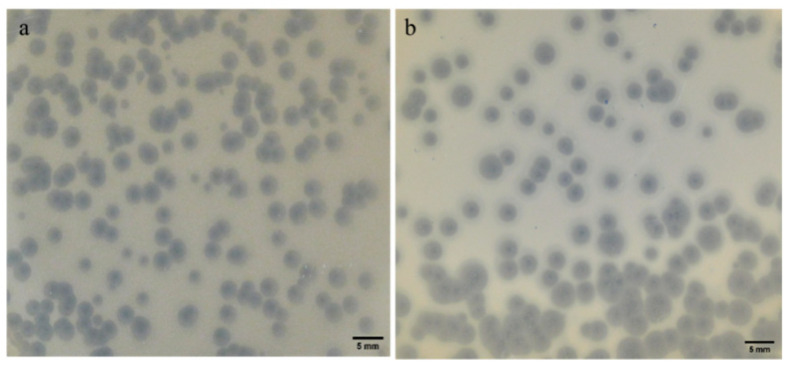
Plaque morphology for the new isolated phages: (**a**) phage KpTDp1; (**b**) phage KpTDp2.

The plaque morphology and size of both phages were examined (Figure 1). Both phages produced plaques with relatively similar characteristics: a clear lytic zone at the center, indicative of bacterial lysis, surrounded by a turbid halo that may suggest depolymerase activity. Despite these similarities, the plaque sizes differed slightly between the phages. For phage KpTDp1, the average diameter of the lytic zone was 1.267 mm, which was smaller than that of phage KpTDp2, with an average diameter of 2.550 mm. A positive correlation was observed between the size of the lytic zone and the putative depolymerization halo, with KpTDp1 exhibiting a smaller halo (2.574 mm) compared to KpTDp2 (4.281 mm).

### 2.3. Phages Exhibit a Narrow Host Range

The host range of the two isolated phages was assessed by spot-testing against 17 available strains, including members of the ESKAPE group (*S. aureus*, *K. pneumoniae*, *A. baumannii*, and *P. aeruginosa*), as well as other genera and species such as *Escherichia coli*, *Klebsiella oxytoca*, *Salmonella bongori*, *Pseudomonas putida*, *Staphylococcus xylosus*, and *Citrobacter freundii* (Table 1).

Both phages exhibited a narrow host range. Although seven additional *Klebsiella* strains were tested, both phages were able to form clear lytic plaques only on a reference strain of *K. pneumoniae* with capsular type K2, in addition to their original host. Sequencing of the *wzi* gene, a key determinant of capsular type in *Klebsiella* spp., revealed that the original host strain, Kp704, was capsular type K28. This finding suggests that the phages can infect at least two distinct capsular types within *Klebsiella* spp. In addition, both phages showed differential tropism, targeting a *K. oxytoca* and an *S. bongori* strain for KpTDp1 and KpTDp2, respectively.

### 2.4. Lower MOI Enhances Phage Replication

**Figure 2:**
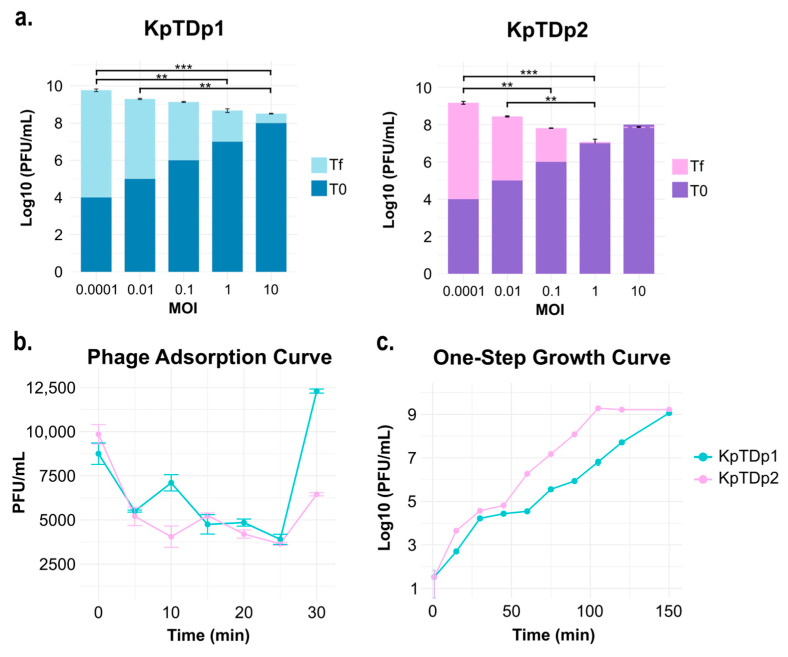
Phage replication dynamics: (**a**) Effect of MOI variation on phage amplification. The dark bars represent the initial phage titer (T0), while light-colored bars show the final phage titer after amplification (Tf). Statistical significance is indicated by ** *p* < 0.01 and *** p < 0.001 (Dunn’s test). Note that for phage KpTDp2 at an MOI of 10, the final titer (dotted pink line) was lower than the initial titer. Error bars indicate the standard error of the mean (SEM). (**b**) Decrease in phage titers due to adsorption to bacteria over 30 min. (**c**) One-step growth curve showing phage titers over 150 min post-infection. Error bars indicate the SEM.

The optimal multiplicity of infection (MOI) may be considered as the ideal ratio of phage particles to bacterial cells that results in the highest phage progeny after amplification. To test the replication ability of phages at different MOIs, bacterial suspensions containing 10^7^ CFU/mL were mixed with phage suspensions ranging from 10^3^ to 10^8^ PFU/mL. We observed an inverse correlation between MOI and replication ability for both phages (Figure 2). Lower initial phage titers resulted in higher final replication titers for KpTDp1 (*p*-value < 0.01; Kruskal–Wallis test). While the titer increased by more than 5 logs when infecting at an MOI of 0.0001, it increased by less than 1 log when infecting at an MOI of 10. This demonstrates that an excess of phage may hinder replication, likely due to the rapid depletion of available bacteria for replication. The same trend was observed for KpTDp2 (*p*-value < 0.01; Kruskal–Wallis test), where infecting at an MOI of 1 already hampered phage replication (Figure 2a).

We further characterized phage replication at low initial MOIs (approximately 0.0001–0.001). Phages adsorbed rapidly to bacteria, as indicated by a sharp decrease in titer within the first 5 min. Adsorption continued over the subsequent 20 min, coinciding with the latent period, and reached maximum adsorption at 25 min post-infection (55.4% for phage KpTDp1 and 62.9% for phage KpTDp2) (Figure 2b). Thereafter, phage titers increased exponentially, with KpTDp2 reaching the stationary phase earlier (105 min post-infection) than KpTDp1 (150 min post-infection) (Figure 2c).

### 2.5. Lower MOI Prolongs Phage Control of Bacterial Growth

**Figure 3:**
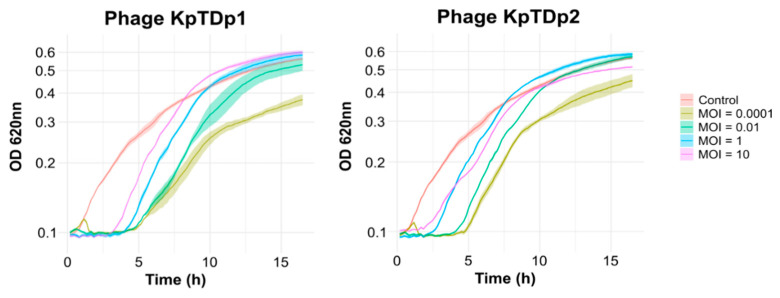
Growth curves of phage-infected bacteria at different MOIs. The control represents the bacterial culture Kp704 without phage. Shaded areas indicate the standard error of the mean (SEM).

We next assessed whether the MOI influences the ability of phages to control bacterial population growth. Liquid infections were conducted at varying initial MOIs, and the optical density at 620 nm (OD_620_) was monitored over 16.5 h. Both phages effectively lysed bacterial cultures across all tested MOIs (Figure 3). An inverse correlation between MOI and the phages’ killing efficacy was observed. At an MOI of 0.0001, the bacterial culture initially grew until phage replication surpassed the bacterial growth rate, leading to a sharp decrease in OD. This condition proved to be the most effective, with phages suppressing bacterial growth for up to 5 h post-infection (hpi) before resistant bacteria emerged. At higher MOIs, phages also significantly inhibited bacterial growth. However, the emergence of resistant bacteria occurred more rapidly compared to the lowest MOI.

### 2.6. Temperature and pH Tolerance of Phages

**Figure 4:**
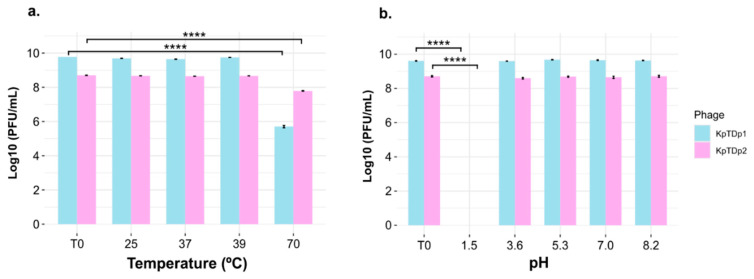
Stability of phages KpTDp1 and KpTDp2 across different conditions. (**a**) Temperatures; (**b**) pH. The initial titer is represented as T0. Error bars indicate the standard error of the mean (SEM) (**** *p* < 0.0001; Tukey’s HSD test).

The stability of both phages was evaluated under different temperatures and pH conditions. Phage titers remained stable after one-hour exposure to 25 °C, 37 °C, and 39 °C. However, a significant reduction in titer was observed following incubation at 70 °C (*p*-value < 0.0001; Tukey’s HSD test), although the viral suspension was not completely degraded (Figure 4a). Phages exhibited high stability across pH values ranging from 3.5 to 8.2. However, at a highly acidic pH of 1.5, phage viability dropped drastically, with no detectable phage particles remaining (*p*-value < 0.0001; Tukey’s HSD test) (Figure 4b).

### 2.7. Genomic Features and Lifestyle Prediction of KpTDp1 and KpTDp2

For further characterization of the two isolated phages KpTDp1 and KpTDp2, whole genomes were sequenced. The analysis revealed that both phages were double-stranded linear DNA genomes. The genome of KpTDp1 was 49,311 bp in length with a G + C content of 48.5%, while the genome of KpTDp2 was 49,084 bp long and has a G + C content of 50.7%. Structural gene annotation revealed 66 open reading frames (ORFs) for KpTDp1, with 28 encoded on the negative strand and 38 on the positive strand. For KpTDp2, 75 ORFs were predicted, 58 of which are encoded on the negative strand and 17 on the positive strand.

The lifestyle prediction indicated that both phages are most likely lytic. PhaTYP showed virulence percentages exceeding 0.999 for both phages. However, PhageAI predicted a lytic cycle of 77.75% for KpTDp1 and 76.96% for KpTDp2.

### 2.8. Taxonomic and Phylogenetic Analysis

**Figure 5:**
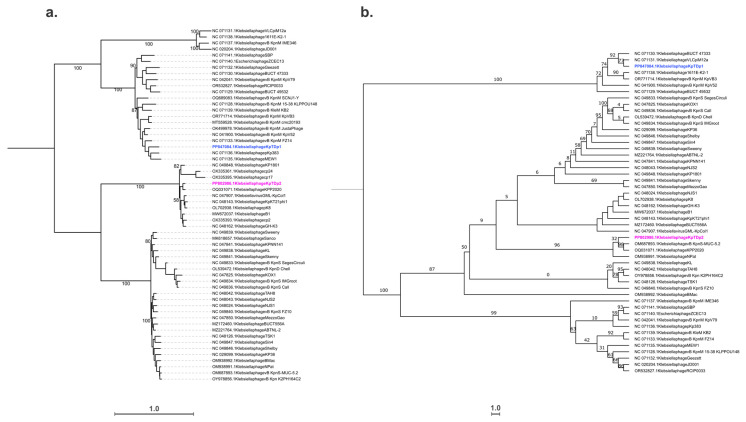
Phylogenetic tree generated with IQ-TREE: (**a**) Phylogeny constructed using the complete genomes of the two newly identified phages, KpTDp1 (blue) and KpTDp2 (pink), along with the genomes of 56 members of the genera *Jedunavirus* and *Webervirus*. (**b**) Phylogeny created using the amino acid sequence of the conserved gene encoding the large terminase subunit. Ultrafast bootstrap support values are shown at the nodes for both trees. The scale bar represents the number of substitutions per site.

To perform a phylogenetic analysis, whole-genome sequences were aligned using MAFFT, and the tree was generated with IQ-TREE. The degree of similarity was analyzed among the 58 input sequences, including the two phages under study and members of the two potential genera, *Jedunavirus* and *Webervirus*, to which they are most closely related. The resulting phylogenetic tree confirmed that the two phages belong to different genera, as they grouped into distinct clades (Figure 5a). A closer examination revealed that phage KpTDp1 clusters within the clade of the *Jedunavirus* genus, potentially representing a transitional cluster to a new, undescribed genus. However, the bootstrap values were insufficient to support a more definitive classification. For phage KpTDp2, it grouped with phages classified within the *Webervirus* genus. The phylogenetic tree indicated that this genus comprises two major clades, suggesting the potential reclassification of *Webervirus* into two distinct subgenera.

A second phylogenetic tree was constructed using a conserved gene, the large terminase subunit, to reconstruct the evolutionary pathway. Amino acid sequences were aligned with Clustal Omega, and the tree was generated with IQ-TREE. These results confirmed the classification of both phages into distinct genera (Figure 5b). However, the bootstrap values were insufficient to support a more detailed classification within the clades.

### 2.9. Functional Annotation

**Figure 6:**
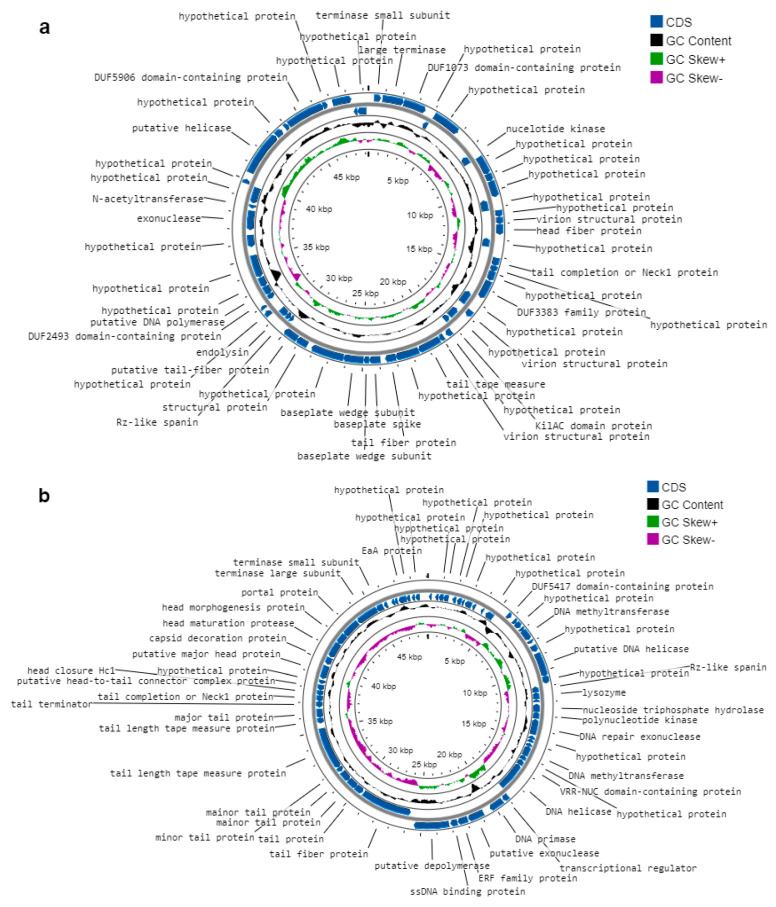
Circular genomic maps and annotation of phages. (**a**) KpTDp1; (**b**) KpTDp2. Genomic maps were generated using the CGView tool (https://cgview.ca/, accessed on 25 November 2024).

Functional annotation of the ORFs was performed using BLASTX against non-redundant protein databases. For KpTDp1, 63.64% of the ORFs could be assigned functions based on homologies with known sequences, while for KpTDp2, 52% of the predicted genes had identifiable functions. The remaining genes corresponded to hypothetical proteins with unknown functions (Figure 6).

In the KpTDp1 genome, all structural and assembly genes were located on the positive strand, in contrast to KpTDp2, where these genes were found on the negative strand. For genes involved in DNA metabolism, including DNA polymerase, helicase, primase, transcriptional regulators, methyltransferase, nucleotide kinase, and others, the predicted genes were almost equally distributed across both strands. A full list of the predicted ORFs, their orientations, and functions is provided in Table S1. Among the 66 ORFs in the KpTDp1 genome, ORF 11 showed the lowest homology in BLAST analysis with known sequences in the databases, though it likely encodes a nucleotide kinase based on a 72.16% amino acid identity. Additionally, ORF 54 was notable for its 100% identity with the amino acid sequence of N-acetyltransferase, which was previously described only in *Klebsiella* phage vB_KpnM_IME346 [17].

All sequences predicted as host lysis proteins demonstrated strong homology and amino acid identity with known sequences in the databases. In both phages, host lysis is mediated by four ORFs—three on the negative strand and one on the positive strand. Functional annotation suggests that both phages likely produce spanins to disrupt the outer membrane during the final stages of host lysis. However, while KpTDp1 contains two-component spanins, KpTDp2 encodes a unimolecular spanin [18]. In addition to the predicted spanins encoded by ORF 40 and ORF 41, KpTDp1 also produces an endolysin (ORF 42), which plays a role in the release of viral progeny [19]. Although no depolymerase was predicted for KpTDp1, ORF 45 in the KpTDp2 genome likely encodes a depolymerase, with over 99% amino acid identity to known sequences. Additionally, ORF 29 in KpTDp2 encodes a holin involved in host cell wall degradation, showing 100% amino acid identity with a reference sequence [19].

### 2.10. Comparison of KpTDp1 and KpTDp2 Genomes with Closely Related Phages

**Figure 7:**
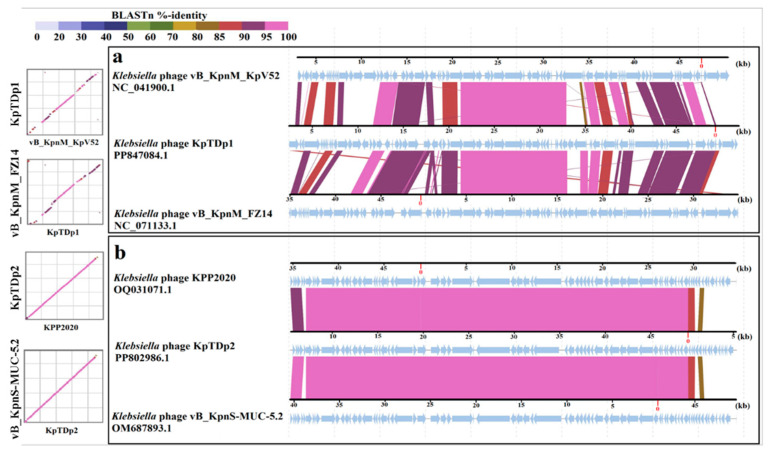
Comparison of phage genomes KpTDp1 and KpTDp2 with closest relatives: (**a**) KpTDp1 aligned with vB_KpnM_KpV52 (NC_041900.1) and vB_KpnM_FZ14 (NC_071133.1). (**b**) KpTDp2 aligned with KPP2020 (OQ031071.1) and vB_KpnS-MUC-5.2 (OM687893.1). The arrows indicate the product encoded in each CDS. Color coding indicates the percentage of similarity between the sequence fraction.

Based on the intergenomic similarity results, *Klebsiella* phages vB_KpnM_KpV52 and vB_KpnM_FZ14 were selected for genomic comparison with the KpTDp1 genome, as they exhibited the highest similarity percentages. Similarly, for KpTDp2, the genome comparison was performed using DIGAlign with *Klebsiella* phages KPP2020 and vB_KpnS-MUC-5.2, which showed the closest genomic similarities (Figure 7).

When comparing KpTDp1 with *Klebsiella* phage vB_KpnM_KpV52, the overall gene arrangement showed a general correlation, with slight shifts in a few base pairs in most cases. Genes encoding the same functions were found at nearly identical loci, with BLASTN identity percentages varying between 85% and over 95%. The highest similarity was observed in structural and assembly genes (up to 97%), followed by DNA metabolism genes. The lowest identity percentage (82.6%) was observed in the DNA polymerase coding region. The lysis genes exhibited a high percentage of identity (96.5%), while the KpTDp1 and *Klebsiella* phage vB_KpnM_FZ14 showed a high level of identity (over 85%). However, the positions of similar genes were not conserved.

In the case of KpTDp2, comparison with the two phages KPP2020 and vB_KpnS-MUC-5.2 resulted in better alignment. Similarities in most genes exceeded 95%. Only a very small region, comprising hypothetical proteins, gave a BLASTN identity of 82%. The highest percentage of identity was observed for structural and assembly genes and those involved in the lysis process (up to 98%).

## 3. Discussion

Phage research and development of phage-derived therapeutics is of special interest, particularly in the fight against antibiotic resistance [20,21]. Here, we described two novel phages, KpTDp1 and KpTDp2, isolated using a Tunisian clinical strain of *K. pneumoniae* as host, with lytic activity against capsular types K2 and K28. Interestingly, these capsular types are associated with high antibiotic resistance and considered among the most virulent strains of the species [22]. Notably, only a limited number of phages targeting K28 strains have been reported [23]. The only other *K. pneumoniae* phage isolated in Tunisia, TUN1, is specific to capsular type K64 [16], associated with a high risk of clone spread worldwide. Given the lytic activity of these new phages against K2 and other capsular types, KpTDp1 and KpTDp2 are suggested as potential therapeutic agents to combat hypervirulent *K. pneumoniae* strains, especially considering that the K2 capsular type is involved in 42.9% of invasive infections [22].

The limited host range is a common characteristic of *Klebsiella* phages, which is thought to be influenced by the number and types of depolymerases they encode [24]. Most *Klebsiella* phages are restricted to infecting one or a few capsular types [23]. This is the case of phages KpTDp1 and KpTDp2, which infected K2 and K28 *K. pneumoniae* strains. However, some phages, such as ΦK64-1, encode multiple depolymerases, enabling them to target up to 10 different capsular types [25]. A recent study isolated phage vB_Kpn_K30λ2.2, which demonstrated the ability to infect 23 different capsular types of *Klebsiella* spp. in semi-solid medium, making it one of the phages with the broadest known host range [26]. In addition, KpTDp1 and KpTDp2 also showed the ability to target and infect a *K. oxytoca*, and a *S. bongori* strains, respectively. Other phages have already been described with tropism for different bacteria genera [27,28,29], and this may be possible due to the relatively essential high conservation of structural antigens such as LPS, CPS, or outer membrane proteins [30].

The haloes observed around the lytic plaques of KpTDp1 and KpTDp2 suggested that these phages may encode depolymerases with capsule-degrading activity [25]. These haloes result from the depolymerization caused by these enzymes, which, unlike the infective viral particles, cannot induce bacterial lysis and act only at the surface level [31]. Gene annotation confirmed that phage KpTDp2 encoded a putative depolymerase in ORF 45 similar to that of phage NPat. However, no gene with predicted depolymerase activity was identified in the genome of phage KpTDp1. More detailed analysis is required to assess whether the receptor-binding proteins of these phages have potential depolymerase activity. Currently, tools such as DepoScope [32], Phage DPO [33], and DePP [34] are available to facilitate such investigations. Additionally, at least four proteins involved in bacterial lysis were predicted for both phages. Spanins, which facilitate the disruption of the bacterial outer membrane during phage particle release, were identified [19]. Phage KpTDp2 appears to employ a unimolecular spanin, whereas KpTDp1 encodes two spanin-like proteins, suggesting a two-component spanin system [18]. Furthermore, KpTDp1 encodes an endolysin with high homology to that described for phage KP1 [35]. While no endolysin was annotated in the genome of KpTDp2, it does encode a holin identical to that of phage vB_KpnD_PeteCarol. Together, these lysis proteins form diverse arsenal enabling phages to lyse bacterial cells efficiently, presenting potential opportunities for phage-based applications.

The experiments conducted in this study demonstrated that both phages, KpTDp1 and KpTDp2, were more efficient at low MOI, exhibiting better lysis and replication rates. Similar findings were reported for other *K. pneumoniae* phages, including K9w5, GH-K3, and CM_Kpn_HB132952, where low MOI was also found to be optimal for phage replication [36,37]. This suggested that the cautious exploitation of the available cellular resources may be a better strategy to increase viral production.

Remarkably, both phages showed high stability when subjected to temperature fluctuations, tolerating variations up to 70 °C for at least one hour. This stability is particularly relevant for logistical considerations and transport. Similarly, other phages, such as KP1, ZCKP2, and VTCCBPA43, have been shown to retain their lytic activity even at 80 °C [35,38]. Future studies should investigate the stability of these phages at low temperatures to evaluate their suitability for long-term storage. The phages also exhibited considerable stability under pH variations. KpTDp1 and KpTDp2 maintained their lytic activity at pH values ranging from 3.5 to 8.2, though exposure to an extremely acidic pH (pH = 1.5) was lethal for both. Similar findings have been reported for phages vB_KpP_HS106, IME268, and KP12 [35,39,40]. This pH tolerance makes these phages promising candidates for applications in environments with varying pH levels, such as the human body, and for potential use in combination with other chemical compounds [41].

Although these new phages share many similar features, genomic analysis confirmed that they belong to two distinct genera with no similarities. The results indicated that KpTDp1 showed the highest similarity to phage vB_KpnM_KpV52, which was classified within the *Jedunavirus* genus. However, the similarity between KpTDp1 and the other 22 members of this genus was less than 70%. Based on this finding, KpTDp1 could represent a new viral genus. However, the lack of information regarding the closest 22 members of the *Jedunavirus* genus complicates the proper characterization of this phage based on its closest relatives. The intergenomic analysis of KpTDp2 confirmed its membership in the *Webervirus* genus, although it was not able to be assigned to a specific species. Indeed, the intergenomic similarity with the two phages, KPP2020 and vB_KpnS-MUC-52, was lower than 94.2%. These two phages could potentially represent a single species within the *Webervirus* genus, making them the closest known species to KpTDp2. Additionally, the phylogenetic tree constructed revealed a division of the members of the *Webervirus* genus into two large branches. This observation suggested that the genus may warrant reclassification.

The high specificity and stability of these phages, combined with their predicted lytic lifestyle, make them excellent candidates for phage therapy. Their high specificity allows for the development of tailored therapies targeting only pathogenic strains, thereby minimizing the risk of dysbiosis and preserving commensal bacteria [42]. However, phage production for therapeutic purposes can be very costly, making single-phage treatments economically challenging. This limitation can be mitigated through the use of phage cocktails, which combine two or more phages to increase the spectrum of activity [43]. Further efforts are needed to monitor the circulation of multidrug-resistant *Klebsiella* spp. in Tunisia and to establish phage banks that cover the most prevalent sequence types of *Klebsiella* spp.

## 4. Materials and Methods

### 4.1. Bacterial Strain

The bacterium used as primary host for phages is a clinical *K. pneumoniae* strain 704 (Kp704) obtained from the Regional Hospital’s Biology Department of Ben Arous, Tunisia in 2019. The isolate, resistant to ticarcillin, ticarcillin/clavulanate, and piperacillin, was cultured in Luria–Bertani (LB) broth medium and stored in glycerol (20%) stocks at −70 °C and at −20 °C for short-term use.

### 4.2. Determination of the Capsular Type

The isolate was genetically identified as *K. pneumoniae* K28 type by PCR amplification of the 16S rRNA gene using a Kit from Thermo Scientific (Waltham, MA, USA), followed by wzi gene PCR amplification for capsule typing [44,45].

### 4.3. Phage Isolation

The wastewater used for phage isolation was collected from sewers in different regions of the Tunis area. The samples were first centrifuged at 4000× *g* for 5 min, followed by a double filtration using a 0.45 then 0.2 µm syringe filter (Minisart^®^ Syringe Filter, Sartorius, Goettingen, Germany). Eight hundred microliters from the filtrated were inoculated with two hundred microliters of an overnight bacterial culture and left at room temperature for 15 min. The mixture was added to 3.5 mL of 0.7% semi-solid LB medium containing 3 mM CaCl_2_, poured into a dish containing 1.5% solid LB medium supplemented with CaCl_2_, and incubated at 37 °C for 18 h [31]. An isolated and distinct lysis range was selected and then collected in 50 µL of medium. Centrifugation at 14,000× *g* was then carried out to recover 30 µL of supernatant, of which 10 µL was used to reinfect the host culture in plates by the double agar method previously described. This step was performed three times to ensure the purity of obtained phages [46].

### 4.4. Phage Amplification and Titration

Phage amplification was performed by inoculating 980 µL of LB medium with 10 µL of bacterial culture in stationary phase, and 10 µL of phage suspension having been obtained following re-suspension of a single lysis range in LB medium. The mixture was incubated at 37 °C for 2.5 h with shaking at 700 rpm and then centrifuged at 14,000× *g* for 10 min. Eight hundred microliters of supernatant were then recovered, aliquoted in Eppendorf tubes and stored at −70 °C [31].

The titration of phage suspensions was performed by preparing a series of dilutions from amplified tubes (10^−2^ to 10^−8^). These were considered to infect their respective hosts and grown in plates by the double agar method previously described. After 24 h of incubation at 37 °C, the lysis plates were counted, the dilutions of which allowed for the calculation of the phage concentration [47].

### 4.5. The Multiplicity of Infection (MOI)

The host bacteria were incubated overnight at 37 °C with shaking, followed by infection with various concentrations of phage. An equal volume of bacterial suspension (10^7^ CFU/mL) was maintained to obtain various MOI values (10, 1, 0.1, 0.01, and 0.001). In order to compare the efficacy of the different MOI values tested, amplification was carried out as follows: the mixture was incubated at 37 °C for 2.5 h with shaking. The phage titer of each MOI was determined using the double agar method. This process was carried out three times and the titers of each MOI were recorded [48].

### 4.6. Host Range Determination

The infectivity of the isolated phages was tested using the spot test method on 17 bacterial strains from the ESKAPE group (*K. pneumoniae*, *S. aureus*, *A. baumannii*, and *P. aeruginosa*) or others (*E. coli*, *K. oxytoca*, *S. bongori*, *P. putida*, *S. xylosus*, and *Citrobacter freundii*) in order to establish their host spectrum. Two microliters of the phage stock were deposited on the surface of soft agar previously inoculated with one of the bacterial strains. After incubation at 37 °C for 24 h, the absence of growth in the deposition area or the presence of turbidity in the zone showed that the phage was able to recognize and affect the tested bacteria. The experiment was carried out in triplicate [49].

### 4.7. Effect of Phages on Bacterial Growth

The optical density of the bacterial culture in the presence of a concentration of 10^5^ phage suspension was measured in real time every 10 min for 16.5 h. Growth curves plotted from the plate reader were used to assess the effect of phage suspension on bacterial growth, in comparison with control growth curves. The experiment was carried out in triplicate [50].

### 4.8. Phage Temperature and pH Stability

The stability of the two isolated phages was studied by incubating 100 µL of a phage stock (10^9^ for KpTDp1 and 10^8^ for KpTDp2) for one hour at different temperatures (25, 37, 39, and 70 °C) [51]. The pH of SM buffer (200 mM NaCl_2_, 10 mM MgSO_4_, and 50mM Tris-HCl, pH 7.5) was adjusted to 1.5, 3.5, 5.0, 7.3, or 8.0 by adding HCl or NaOH. Ten microliters of stock phage solution were then added to 990 µL of SM buffer and incubated at 37 °C for one hour. Phage titration after heat treatment or pH variation was carried out using the double agar method described above. All the conditions and steps in this experiment were carried out in triplicate.

### 4.9. Adsorption and One Step Growth Curve

In phage adsorption assays, the bacterial culture and phage suspension were mixed at MOI = 0.0001 and incubated at 37 °C with shaking. Aliquots of 1mL were collected every 5 min for 30 min, centrifuged, filtered through a 0.22 µm filter, and titrated using the double-layer agar method. The decrease in the number of free phages indicates the rate of adsorption [40].

To determine the latent period, burst time period, and burst size, bacterial culture was mixed with phage suspension at MOI = 0.0001. After 15 min of incubation at 37 °C without shaking, the mixture was centrifuged at 13,000× *g* for 10 min. The pellet was suspended in 100 mL of LB medium and incubated at 37 °C with shaking. Aliquots of 1 mL were taken every 15 min for 150 min and titrated using the soft agar overlay method. The latent period is the timing of phage-induced host cell lysis and is determined from the curve as the interval between adsorption and host lysis. The burst size is the average number of phages released per infected bacterial cell and corresponds to the ratio of the final phage titer to the number of initial infected bacterial cells. The burst time period is the interval between the latent period and the stationary phase of the phage titer [52].

### 4.10. DNA Isolation and Sequencing

DNA extraction was initiated by further amplification of the phage by inoculating 6 mL of LB medium with 100 µL of phage suspension and 100 µL of an overnight bacterial culture. The mixture was incubated for 2.5 h at 37 °C with 200 rpm agitation before centrifugation at 4000× *g* for 15 min. The supernatant was then collected in a new tube, filtered with a 0.22 µm filter and ultra-centrifuged for 2h at 40,000× *g*. The pellet was re-suspended in 200 µL of SM buffer and titrated. Phage DNA extraction and purification were performed in six steps using the Genomic DNA Clean & Concentrator Kit from Zymo Research. Phages were first diluted 10-fold in Turbo DNAse 1× buffer (100 μL final volume) and then 2 μL of Turbo DNAse and 1 μL of benzonase were added. The digest was incubated for 1 h at 37 °C, and 1 μL of DNase Turbo was added for another hour. To inactivate the DNases, 0.5M EDTA was used and the mix incubated at 75 °C for 10 min. To digest phage capsids, 10 μL of 10% SDS and 5 μL of proteinase K were added and the mix incubated for 45 min at 55 °C. Before sequencing, quantification of phage DNA was performed by qubit DNA quantification. Sequencing libraries were prepared using the Illumina Nextera XT DNA kit and paired-end reads (2 × 250 bp) were generated in the Illumina MiSeq platform (MiSeq Reagent Kit v2).

### 4.11. Genome Assembly, Annotation, and Bioinformatics Analysis

Phage genomes were assembled using SPAdes version 3.15.4 with the only assembler option. After analyzing and removing host contaminants and other sequencing, a contig with high k-mer coverage per sample was obtained. This contig was considered as the phage genome and coverage was obtained by matching all reads to the contig. In order to predict the genus to which the isolated phages should belong, the assembly generated was compared on the basis of sequence homology with known phage sequences in NCBI via BLASTN. Structural annotation and the coding sequences (CDSs) in each genome were predicted using the GeneMarkS tool (https://genemark.bme.gatech.edu/genemarks.cgi/, accessed on 15 September 2023). Each predicted gene was then annotated by performing a search in the NCBI non-redundant protein sequences (NR) and CDD databases using the basic local alignment search tool (BLAST, https://blast.ncbi.nlm.nih.gov/, accessed on 18 September 2023).

The lifestyle of each phage was predicted using different tools: PhageAI [53] and PhaTYP [54]. For taxonomic classification, phage genomes were compared to the closest relatives found in the NCBI database. The intergenomic similarity was calculated using VIRIDIC software (https://rhea.icbm.uni-oldenburg.de/viridic, accessed on 3 November 2023). For the phylogenetic analysis, whole-genome sequences were aligned using MAFFT [55], and the aminoacidic sequences of the large terminase subunit were aligned using Clustal Omega [56]. Trees were finally constructed with IQ-TREE [57] and plotted using FigTree (http://tree.bio.ed.ac.uk/software/figtree/, 3 November 2023). DIGAlign (https://www.genome.jp/digalign/, 3 November 2023) was used to compare genomes [58,59].

### 4.12. Data Availability

The genetic sequences acquired during this study have been deposited in the database of the National Center for Biotechnology Information database as SRAs under accession numbers SRS21328276 and SRS21289639 generating BioProjects under accession numbers PRJNA1112778 and PRJNA1111440, corresponding to phage KpTDp1 and KpTDp2, respectively. The biosamples were registered and correspond to the respective accession numbers SAMN41434463 and SAMN41391750. The phage genomes were submitted to BankIt under the respective accession numbers PP847084 and PP802986.

## 5. Conclusions

In this study, we isolated and characterized, biologically and genomically, two new phages, KpTDp1 and KpTDp2, that target a clinical isolate of *K. pneumoniae*. Their ability to lyse the capsular type K2 of *K. pneumoniae*, considered one of the most virulent serotypes, makes these phages potential candidates for antibacterial control. The stability and lytic activity of these phages have been well demonstrated, and relevant proteins with potential therapeutic use have been identified through genomic analysis. Despite being isolated from the same environmental source using the same host strain, these phages are classified in different genera. Further research is required to enhance our understanding of their mechanisms of action and explore other potential applications.

## Figures and Tables

**Table 1:** Bacterial strains and host range of KpTDp1 and KpTDp2.

Bacteria	KpTDp1	KpTDp2
*K. pneumoniae* 704 (clinical strain)	+	+
*K. pneumoniae* 156 (clinical strain)	−	−
*K. pneumoniae* 443 (clinical strain)	−	−
*K. pneumoniae* 636 (clinical strain)	−	−
*K. pneumoniae* 754 (clinical strain)	−	−
*K. pneumoniae* 956 (clinical strain)	−	−
*K. pneumoniae* K2 (reference strain B5055)	+	+
*K. oxytoca* (clinical strain)	+	−
*P. aeruginosa* (ATCC 27853)	−	−
*P. aeruginosa* (clinical strain)	−	−
*P. putida* (clinical strain)	−	−
*P. aureus* (ATCC 29213)	−	−
*S. aureus* (clinical strain)	−	−
*S. xylosus* (clinical strain)	−	−
*E. coli* (ATCC 25922)	−	−
*E. coli* (clinical strain)	−	−
*S. bongori* (clinical strain)	−	+
*A. baumannii* 15277 (clinical strain)	−	−
*A. baumannii* 10783 (clinical strain)	−	−
*C. freundii* (clinical strain)	−	−

(+) plaque formation; (−) no plaque formation.

## Data Availability

Data are contained within the article and Supplementary Materials.

## Supplementary Materials

The following supporting information can be downloaded at: https://www.mdpi.com/article/10.3390/antibiotics13121154/s1, Table S1: KpTDp1 and KpTDp2 genome analysis and protein prediction.
